# Supplementary material for: A Longitudinal Study of a Multicomponent Exercise Intervention with Remote Guidance among Breast Cancer Patients
Source: Int J Environ Res Public Health. 2020 May 14;17(10):3425. doi: 10.3390/ijerph17103425 (PMC7277866; doi:10.3390/ijerph17103425)
Supplement: Supplementary file 1 [file ijerph-17-03425-s001.pdf]

Supplementary Table 1: Quality of life, Physical Activity (PA) Estimates, Muscle strength and Cardiopulmonary endurance at 12 weeks.

Variables	Group	12 weeks Mean (SD)	Variables	Group	12 Weeks Mean (SD)
SF-36					
PF	ME	86.52 (8.04)	SPSDCT	ME	19.35 (4.53)
	UC	85.95 (11.36)		UC	16.14 (2.97)
RP	ME	58.70 (37.39)	ALT	ME	20.70 (4.04)
	UC	51.19 (42.92)		UC	19.90 (3.69)
BP	ME	76.52 (13.69)	VO2max	ME	52.72 (9.78)
	UC	79.52 (17.17)		UC	45.77 (15.20)
GH	ME	72.43 (17.39)	PA Estimates		
	UC	63.10 (18.46)	Social support	ME	17.39 (5.37)
VT	ME	67.17 (6.18)		UC	14.38 (4.27)
	UC	63.10 (9.15)	PA hinder	ME	23.48 (6.62)
SF	ME	94.57 (17.61)		UC	28.81 (4.40)
	UC	94.05 (21.51)	EA	ME	36.57 (5.69)
RE	ME	75.36 (35.13)		UC	36.24 (2.12)
	UC	69.84 (39.31)	PA enjoyment	ME	18.91 (2.23)
MH	ME	54.09 (4.95)		UC	18.95 (2.22)
	UC	50.48 (5.58)	Self-efficacy	ME	74.57 (19.39)
HT	ME	4.30 (0.97)		UC	57.67 (23.81)
	UC	3.90 (0.83)			

SD, standard deviation; CI, confidence interval; ME, multicomponent exercise group; UC, usual care group; SF-36, the Mos 36-item Short Form Health Survey; PF, Physical Functioning; RP, Role-Physical; BP, Bodily Pain; GH, General Health; VT, Vitality; SF, Social Functioning; RE, Role-Emotional; MH, Mental Health; HT, Reported Health Transition; EA, Expected accomplishments; VO2, max maximal oxygen uptake; SPSDCT, Stand-up and sit-down chair test (number of times standing up from the chair within 30 s); ALT, Arm lifting test (30 s dumb bell of 5 pounds or 2.3 kg lifting test).
